# A Case of Advanced Charcot-Marie-Tooth Disease Showing Extreme Lumbosacral Nerve Root Hypertrophy

**DOI:** 10.7759/cureus.108971

**Published:** 2026-05-16

**Authors:** John Paul Lemchak, Justin Brown-Gnarra, Christian Roberti, Murat Ibatullin

**Affiliations:** 1 Medical School, Lake Erie College of Osteopathic Medicine, Bradenton, USA; 2 Radiology, Lake Erie College of Osteopathic Medicine, Bradenton, USA

**Keywords:** charcot-marie-tooth disease, demyelinating neurological disorder, inherited peripheral neuropathy, pes cavus deformity, radiographic findings

## Abstract

Charcot-Marie-Tooth (CMT) disease is the most common hereditary motor and sensory neuropathy. While diagnosis is typically established through clinical findings, nerve conduction studies (NCS), and genetic testing, imaging manifestations such as nerve root hypertrophy are increasingly recognized. This case report presents a 43-year-old man with lifelong progressive distal weakness who presented with severe lower extremity atrophy, pes cavus deformities, and wheelchair dependence. Neurologic examination revealed marked distal weakness and hyporeflexia, while nerve conduction studies demonstrated severely reduced sural nerve conduction velocity. MRI of the lumbar spine revealed diffuse, symmetric hypertrophy of the non-enhancing lumbosacral nerve roots. Corresponding filling defects were seen on myelography, consistent with hypertrophic demyelinating neuropathy. This case highlights advanced CMT with pronounced nerve root enlargement, a feature that can support diagnosis when genetic testing is unavailable. Recognition of imaging findings helps differentiate CMT from other causes of polyneuropathy.

## Introduction

Charcot-Marie-Tooth (CMT) disease is a heterogeneous group of inherited motor and sensory neuropathies. It is the most common hereditary neuromuscular disorder, with an estimated worldwide prevalence of approximately one in 2,500 individuals. The condition is characterized by slowly progressive distal muscle weakness, sensory impairment, and skeletal deformities, most commonly pes cavus and hammertoes [1]. CMT presents along a spectrum of genetic mutations, from the demyelinating (CMT type 1 (CMT1)) form to the axonal (CMT type 2 (CMT2)) and other intermediate forms [1,2]. This classification is based primarily on nerve conduction velocities (NCVs) and underlying pathophysiology [2].

Demyelinating forms of CMT are defined by uniformly slowed NCVs, reflecting impaired saltatory conduction due to myelin dysfunction. CMT1, the most prevalent subtype, is inherited in an autosomal dominant pattern with a clinical onset typically during childhood or adolescence. The most common genetic cause of CMT1 is duplication of the peripheral myelin protein 22 (PMP22) gene [1,3]. Overexpression of PMP22 disrupts Schwann cell function and myelin maintenance, thereby promoting repeated cycles of demyelination and remyelination. This results in Schwann cell proliferation, endoneurial fibrosis, and progressive nerve enlargement [1]. These structural changes explain both the characteristic electrophysiologic findings and the development of hypertrophic peripheral nerves [2,4].

Several other classes of CMT warrant brief discussion. A more severe demyelinating phenotype, CMT type 3 (CMT3), typically presents in infancy and is characterized by profound conduction slowing and early motor impairment. CMT type 4 (CMT4) represents a genetically heterogeneous group of autosomal recessive disorders that also generally manifest in early childhood. In contrast, hereditary neuropathy with liability to pressure palsies (HNPP) is caused by deletion of PMP22. HNPP is characterized by episodic, trauma-associated demyelinating neuropathies rather than continuous disease progression [5].

Axonal forms of CMT, particularly CMT2, usually present in young adulthood. These subtypes present with relatively normal NCVs with some evidence of axonal degeneration. Mutations in mitofusin-2 (MFN2) are the most common genetic cause of CMT2. X-linked CMT (CMTX) most often results from mutations in the gap junction beta-1 (GJB1) protein, which encodes connexin 32 (Cx32). CMTX usually presents during adolescence and shows intermediate slowing of NCVs. Affected males generally experience more severe clinical manifestations [1,6].

Clinical and electrophysiologic evaluation remains essential in the assessment of CMT. Next-generation sequencing has become integral to the modern diagnostic algorithm, enabling molecular confirmation, prognostic refinement, and inheritance counseling [1,2]. Advances in neuroimaging have further expanded the diagnostic workup, with MRI and ultrasound increasingly recognized for their supportive role [1]. Imaging may demonstrate diffuse nerve and nerve root hypertrophy in patients with advanced disease, providing valuable supportive evidence, particularly when genetic testing is unavailable [1,5]. However, nerve root enlargement is not specific to CMT and may be seen in both inherited and acquired conditions, including chronic inflammatory demyelinating polyradiculoneuropathy (CIDP), neurofibromatosis type 1, and paraproteinemic neuropathy [5]. Imaging, therefore, serves a complementary and adjunctive role, and careful clinical correlation remains essential [2,5].

We present a case of advanced demyelinating CMT with marked lumbosacral nerve root hypertrophy demonstrated on MRI and myelography. This case highlights the utility of imaging in distinguishing hereditary from acquired neuropathies. It also illustrates the classic clinical, electrophysiologic, and morphologic features of long-standing CMT.

## Case presentation

A 43-year-old man presented with a history of progressively worsening lower extremity weakness that began in childhood. Over the years, his symptoms progressed to wheelchair dependence, with daily activities significantly limited by weakness. The patient denied recent illness, toxic exposures, or systemic symptoms, and his review of systems was otherwise unremarkable.

On physical examination, the patient was alert and oriented to person, place, and time. Inspection of the lower extremities revealed marked distal muscle atrophy below the knees, producing a characteristic “wine-bottle” appearance, along with pronounced pes cavus deformities of both feet. His gait was steppage in nature when attempting ambulation with significant external support. Motor examination demonstrated severe weakness in the lower extremities, graded as 1/5 strength throughout. Deep tendon reflexes in the lower extremities were diminished at 1+. No upper motor neuron signs were present.

Laboratory evaluation, including complete blood count, liver function tests, and a comprehensive metabolic panel, was within normal limits. Chest radiography and abdominal ultrasound showed no abnormalities.

Nerve conduction studies (NCS) were obtained and demonstrated markedly reduced sural nerve conduction velocity of 11 m/s (reference ≥38 m/s), consistent with a severe demyelinating polyneuropathy [5].

**Figure 1:**
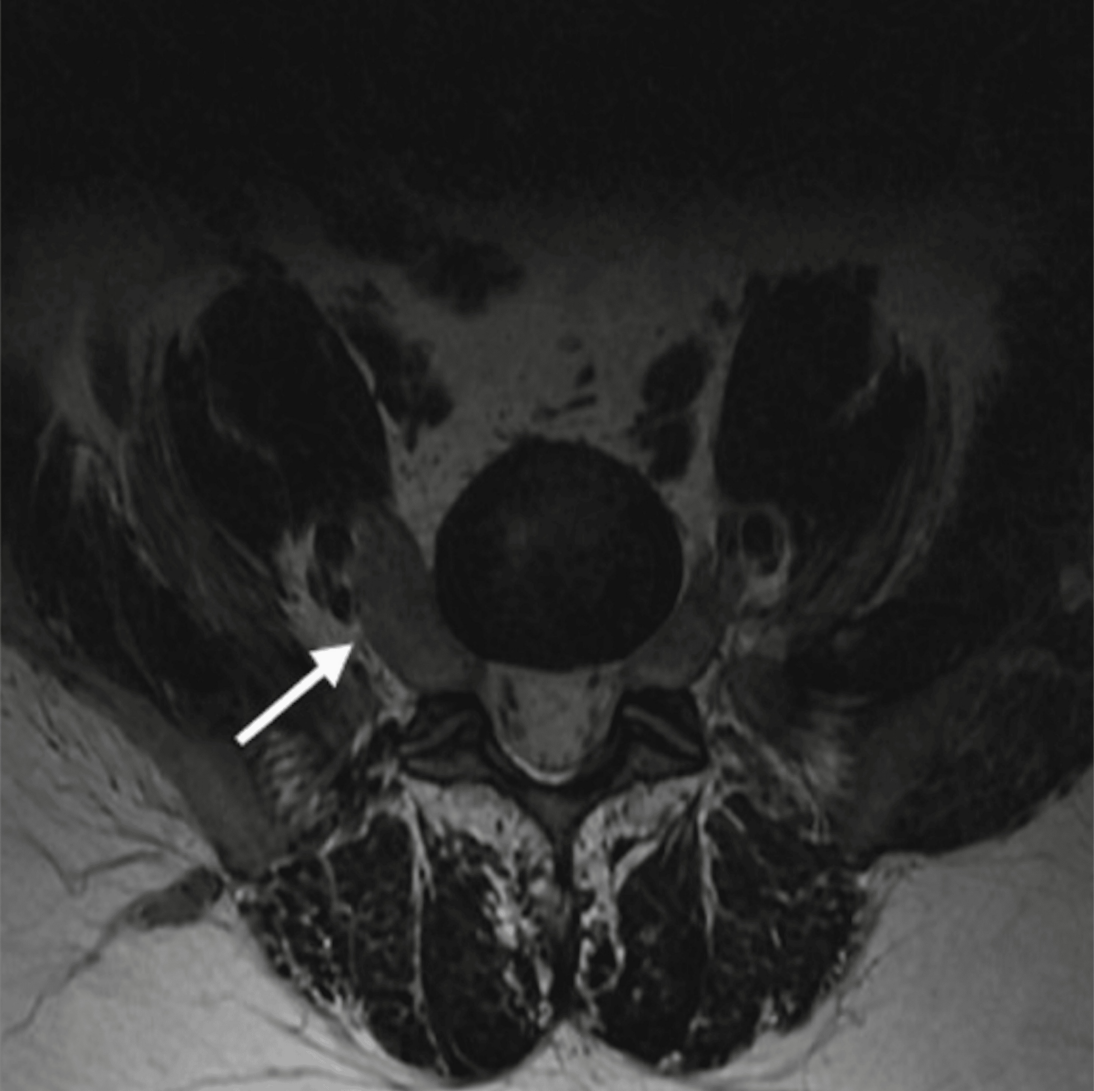
Axial T2-weighted MRI of the lumbar spine Axial MRI demonstrating diffuse symmetric enlargement of the lumbosacral nerve roots without abnormal enhancement. The hypertrophic nerve roots (white arrow) produce a clustered appearance within the intervertebral foramen.

**Figure 2:**
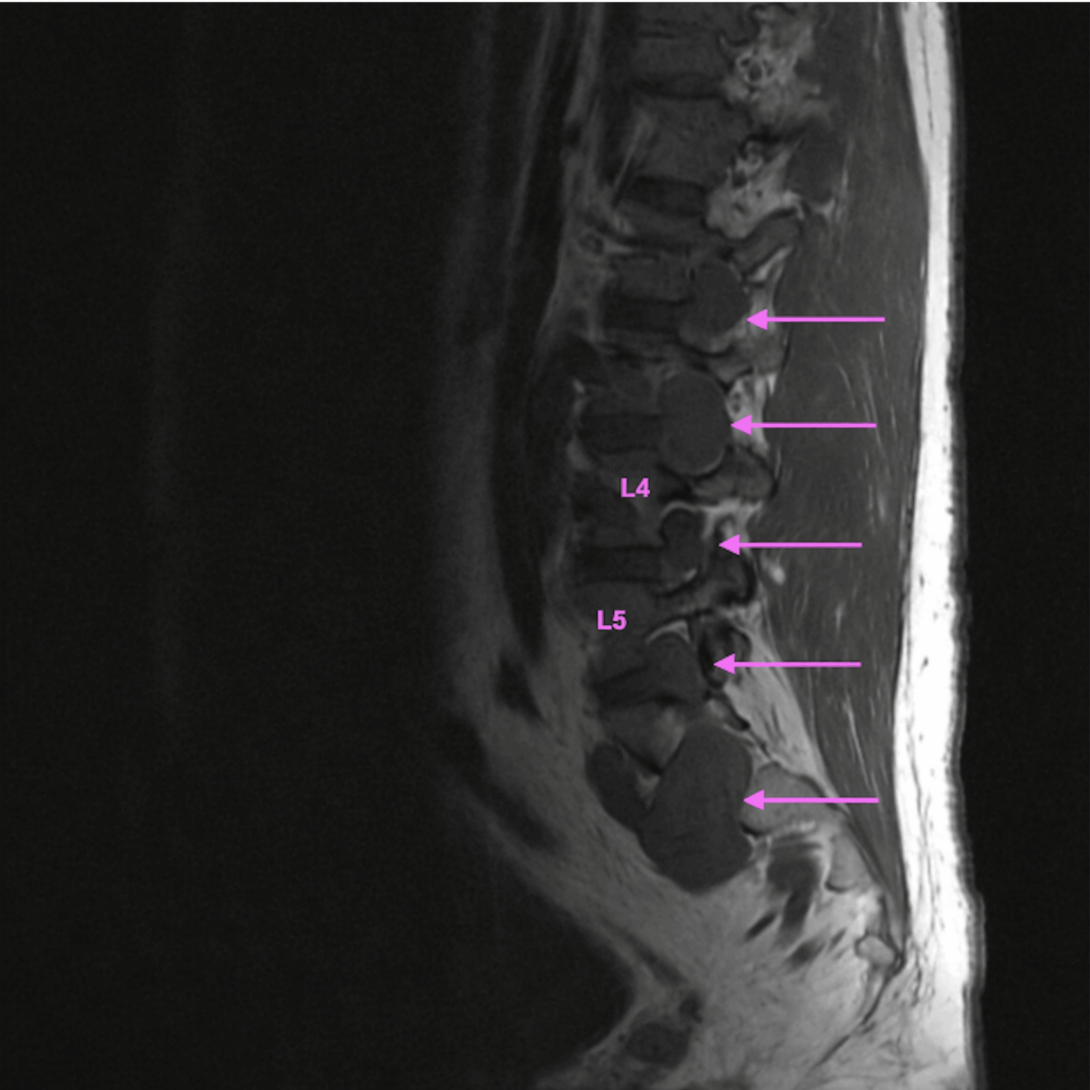
Parasagittal T1-weighted MRI MRI demonstrating marked hypertrophy of non-enhancing lumbosacral nerve roots extending through the neural foramina (purple arrows).

**Figure 3:**
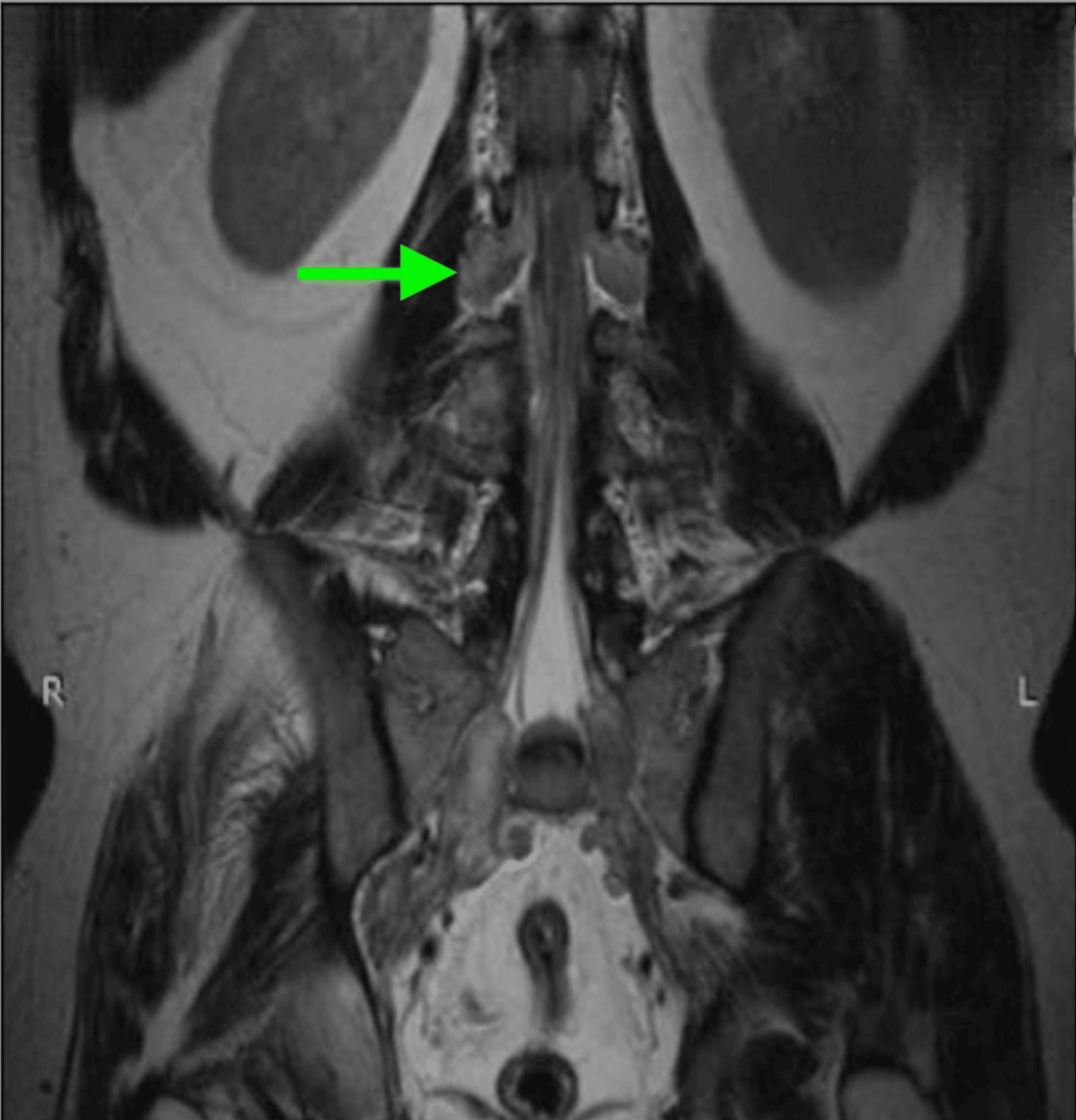
Coronal T2-weighted MRI MRI showing focal enlargement of a lumbar nerve root, consistent with chronic hypertrophic demyelinating neuropathy (green arrow).

**Figure 4:**
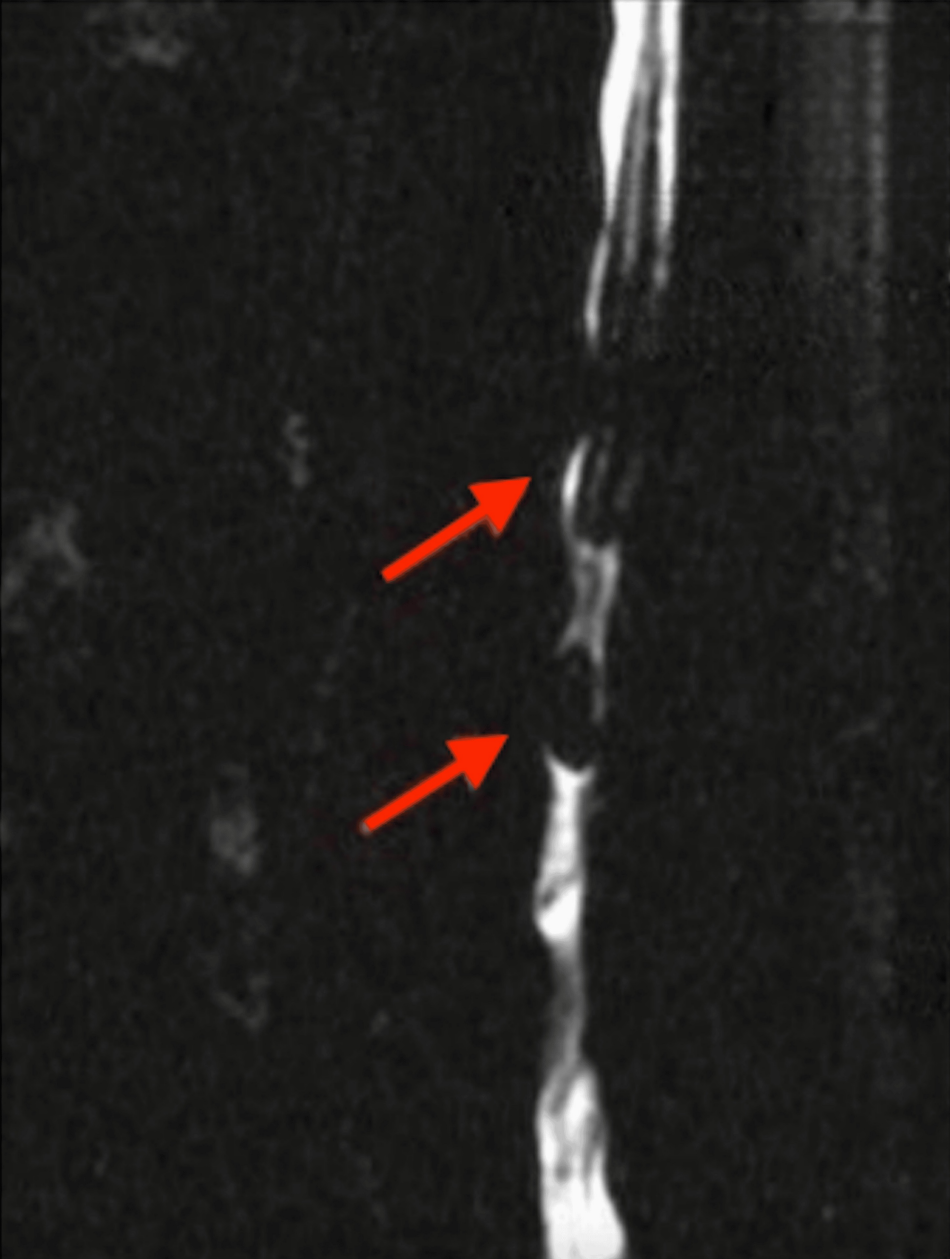
Lumbosacral myelogram Myelogram showing radicular filling defects secondary to nerve root enlargement (red arrows), with additional subtle hypertrophy of adjacent roots suggestive of diffuse involvement of the cauda equina.

MRI of the lumbar spine revealed diffuse, symmetric hypertrophy of the lumbosacral nerve roots without abnormal enhancement (Figures 1-3). Myelography demonstrated radicular filling defects corresponding to enlarged nerve roots, with additional subtle hypertrophy of adjacent roots suggesting generalized involvement of the cauda equina (Figure 4). Taken together, the clinical, electrophysiologic, and imaging findings supported a diagnosis of CMT disease, most consistent with a demyelinating subtype.

## Discussion

The MRI findings in this patient demonstrated diffuse, symmetric hypertrophy of non-enhancing lumbosacral nerve roots with corresponding filling defects on myelography. These findings reflect chronic structural changes consistent with Schwann cell proliferation and endoneurial fibrosis rather than inflammatory or neoplastic processes [1]. Recognition of this imaging pattern is critical, as nerve root hypertrophy is not specific to CMT disease and may be misinterpreted as CIDP, neurofibromatosis, or paraproteinemic neuropathy [5]. When integrated with clinical chronology, childhood onset, skeletal deformities, family history, and uniform electrophysiologic slowing, symmetric non-enhancing nerve root enlargement can provide meaningful supportive evidence for a hereditary etiology [1,2,5].

Based on the clinical presentation, electrophysiologic findings, and imaging characteristics, a comprehensive differential diagnosis was developed to evaluate alternative etiologies. CMT disease, an inherited peripheral neuropathy, was strongly considered given its association with pes cavus and hammertoe deformities [2]. Diagnostic features include distal muscle weakness, muscle atrophy, and markedly decreased nerve conduction velocities, with genetic testing serving as a confirmatory evaluation [2]. Poliomyelitis, an infectious cause of pure motor paralysis, was also considered; diagnosis is established through blood, cerebrospinal fluid, or other cultures demonstrating poliovirus [7]. However, widespread global vaccination efforts and near eradication of the virus make this diagnosis unlikely, though serologic testing or viral cultures could definitively exclude it [7].

Neurofibromatosis type 1, an inherited neurocutaneous disorder characterized by benign nerve sheath tumors and café-au-lait macules, was included in the differential [8]. Although no cutaneous findings were observed on examination, MRI could be utilized to evaluate for underlying neurofibromas. Friedreich’s ataxia, the most common hereditary ataxia, was also considered due to its association with pes cavus and hammertoes [9]. It typically presents in childhood with progressive gait impairment, sensory deficits, and speech dysfunction [9]. Additionally, characteristic MRI findings of neuronal degeneration and dorsal root ganglia hypoplasia were not consistent with this patient’s presentation [9].

Welander distal myopathy, an autosomal dominant disorder characterized by distal upper extremity weakness with later lower extremity involvement, was considered less likely, as abnormal nerve conduction velocities indicate a neuropathic rather than myopathic process [5]. CIDP remains the most clinically important acquired mimic of hypertrophic demyelinating neuropathy and warrants careful exclusion. Unlike CMT, CIDP typically presents with subacute progression, symmetric proximal and distal weakness, areflexia, and potential responsiveness to immunotherapy [10]. The absence of these features, combined with this patient's lifelong disease course and childhood onset, argues strongly against an acquired inflammatory etiology [10]. Toxic peripheral neuropathy was another consideration in the differential diagnosis. In developed countries, this condition is most commonly associated with chemotherapeutic agents or exposure to environmental toxins such as arsenic, lead, or mercury [11]. Toxic neuropathies typically present as length-dependent axonal neuropathies rather than hypertrophic demyelinating disorders [11]. This pattern is inconsistent with the patient's demyelinating electrophysiology, childhood onset, and structural nerve hypertrophy, making a toxic etiology unlikely [11]. Metabolic and nutritional etiologies, including diabetes mellitus and vitamin deficiencies, were also considered. While these conditions can produce distal polyneuropathy, they rarely cause childhood-onset demyelinating neuropathy, making them physiologically inconsistent with the observed phenotype [1,2]. Finally, paraproteinemic polyneuropathy was considered, as abnormal B-cell or plasma cell proliferation can result in excess immunoglobulin production and distal neuropathy [12]. Serum protein electrophoresis and hematologic evaluation could be used to definitively confirm or exclude this diagnosis [12].

This case demonstrates the classic clinical, electrophysiologic, and radiologic manifestations of advanced demyelinating CMT disease, most consistent with CMT type 1. The patient’s lifelong history of slowly progressive distal weakness, severe pes cavus deformities, and symmetric lower extremity atrophy strongly supports a hereditary neuropathy rather than an acquired process. The profound reduction in sural nerve conduction velocity to 11 m/s is characteristic of demyelinating neuropathy [2]. In CMT1, conduction slowing is typically diffuse and uniform across multiple nerves [1,2]. This pattern helps distinguish hereditary demyelination from acquired inflammatory neuropathies such as CIDP, where conduction abnormalities are often patchy and associated with conduction block or temporal dispersion [10]. We acknowledge that only the sural nerve was studied in this case, and a more comprehensive nerve conduction study would have provided greater electrophysiological characterization.

Demyelinating CMT arises from mutations affecting proteins essential for Schwann cell function and myelin stability [3]. In CMT1A, duplication of PMP22 disrupts myelin maintenance and results in repeated cycles of demyelination and remyelination [3]. This process leads to concentric Schwann cell proliferation forming “onion-bulb” structures, segmental demyelination, and progressive endoneurial fibrosis [3]. Over time, these changes produce nerve enlargement that becomes apparent both clinically and radiologically, accounting for the diffuse nerve root hypertrophy observed in this patient [5].

Imaging serves a complementary role in the diagnosis of CMT, particularly in advanced cases or when genetic testing is unavailable or inconclusive. MRI provides visual confirmation of chronic structural nerve changes and aids in distinguishing hereditary neuropathies from inflammatory, neoplastic, or infiltrative processes [5]. Recognition and documentation of these imaging features contribute to the expanding radiologic spectrum of hereditary neuropathies [1]. They also underscore the importance of integrating clinical history, electrophysiologic data, and imaging findings when evaluating chronic polyneuropathy [1].

## Conclusions

This case illustrates advanced demyelinating CMT with marked, symmetric lumbosacral nerve root hypertrophy demonstrated on MRI and myelography. In the setting of a lifelong, slowly progressive neuropathy with characteristic clinical and electrophysiologic features, recognition of non-enhancing nerve root enlargement provides important supportive evidence for a hereditary etiology. Although nerve root hypertrophy is not specific to CMT, its symmetric distribution and chronic appearance can help distinguish hereditary neuropathies from acquired inflammatory, neoplastic, or infiltrative processes. This report reinforces the complementary role of imaging in the diagnostic evaluation of chronic polyneuropathy. It also highlights the importance of integrating radiologic findings with clinical history and NCS. While genetic testing remains the gold standard for definitive diagnosis and subtype identification in hereditary neuropathies, imaging serves an adjunctive role in supporting diagnostic suspicion, particularly in settings where molecular confirmation is unavailable. The absence of genetic confirmation represents a key limitation of this report, and next-generation sequencing and copy number analysis should be pursued whenever hereditary neuropathy is suspected.
